# Supplementary material for: Cooperative herbivory between two important pests of rice
Source: Nat Commun. 2021 Nov 19;12:6772. doi: 10.1038/s41467-021-27021-0 (PMC8604950; doi:10.1038/s41467-021-27021-0)
Supplement: Supplementary file 1 — Supplementary Information [file 41467_2021_27021_MOESM1_ESM.docx]

**SUPPLEMENTARY INFORMATION**

**Cooperative herbivory between two important pests of rice**

Qingsong Liu^1,2,5^, Xiaoyun Hu^1,5^, Shuangli Su^1,5^, Yuese Ning^1^, Yufa Peng^1^, Gongyin Ye^3^, Yonggen Lou^3^, Ted C. J. Turlings^4^, and Yunhe Li^1^*

^1^State Key Laboratory for Biology of Plant Diseases and Insect Pests, Institute of Plant Protection, Chinese Academy of Agricultural Sciences, Beijing 100193, China.

^2^College of Life Sciences, Xinyang Normal University, Xinyang 464000, China.

^3^Institute of Insect Sciences, Zhejiang University, Hangzhou 310058, China.

^4^Laboratory of Fundamental and Applied Research in Chemical Ecology, University

of Neuchȃtel, Neuchȃtel 2000, Switzerland.

^5^These authors contributed equally to this work.

* **Corresponding author**

Yunhe Li, E-mail: [liyunhe@caas.cn](mailto:liyunhe@caas.cn)

**This file contains:**

- **Supplementary Figures 1-4**
- **Supplementary Table 1**

**
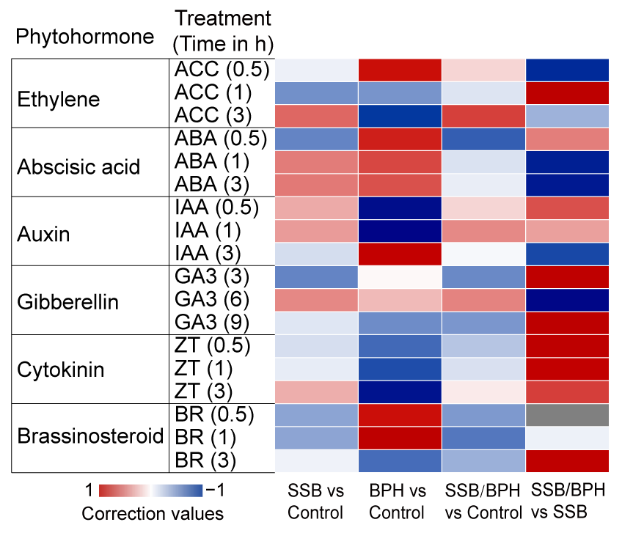
**

**Supplementary Figure 1 Hormonometer analyses for hormone signatures based on transcriptomic responses of rice to herbivory.** The comparisons were made between transcriptomic data generated in rice plants infested by different herbivores and Arabidopsis response to hormone treatments: 1-aminocyclopropane-1-caroxylic acid (ACC; precursor of ethylene); abscisic acid (ABA); indole-3-acetic acid (IAA); gibberellic acid 3 (GA3); zeatin ZT); and brassinosteroid (BR). The colors indicate similarity between herbivore infestation and a particular hormone response (blue and red for negative and positive correlations, respectively, see bottom).

**
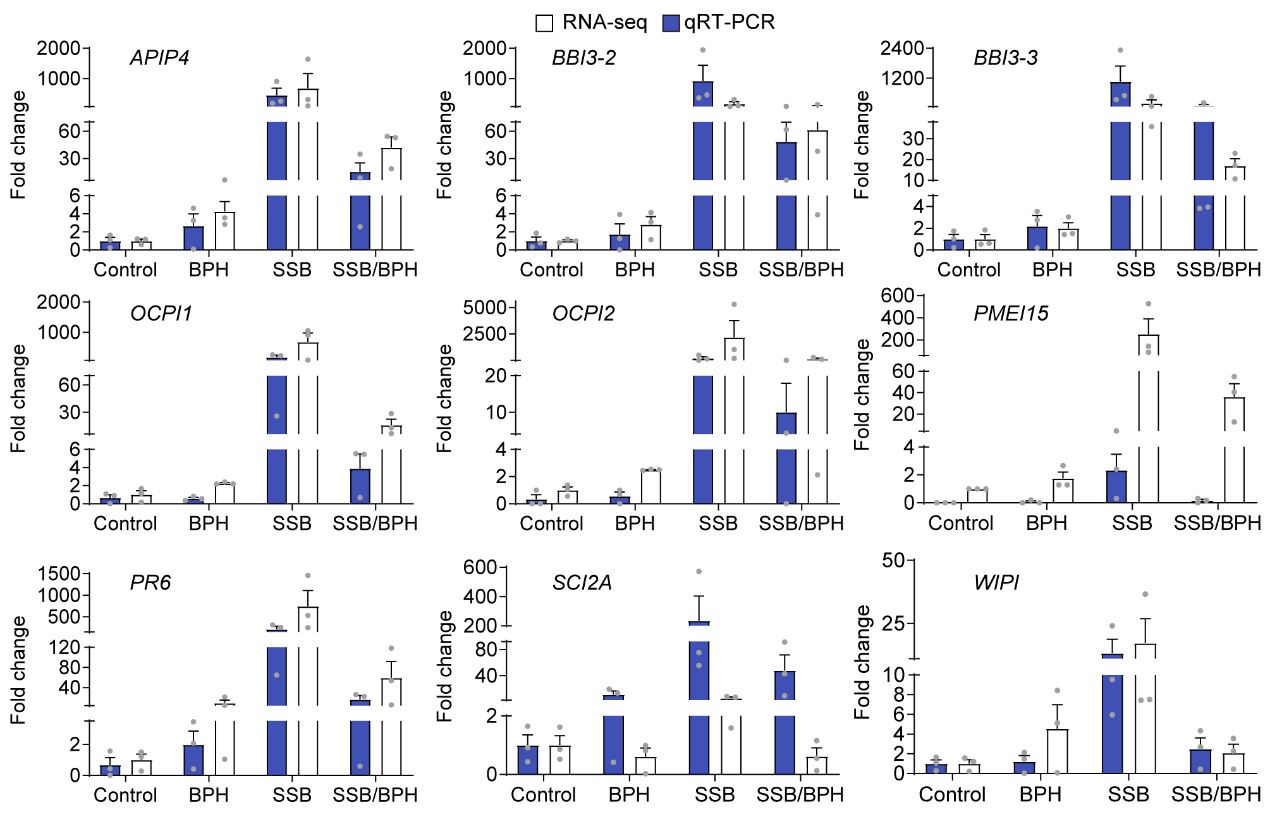
**

**Supplementary Figure 2 Comparison of mRNA expression levels detected by RNA-seq and qRT-PCR.** All qRT-PCR data were normalized against that of the housekeeping gene *ubiquitin 5*. Values are means + SE; n = 3 for both RNA-seq and qRT-PCR experiments. Specific information on these genes is provided in Supplementary Table 1. APIP4, bowman-birk inhibitor AvrPiz-t interacting protein 4; BBI 3-2, bowman-birk inhibitor 3-2; BBI3-3, bowman-birk inhibitor 3-3; OCPI1, *Oryza sativa* chymotrypsin inhibitor-like 1; OCPI2, *Oryza sativa* chymotrypsin inhibitor-like 2; PMEI 15, pectin methylesterase inhibitors 15; PMEI 16, pectin methylesterase inhibitors 16; PR6, pathogenesis-related proteins 6; RASI, rice alpha-amylase/subtilisin inhibitor; SCI2A, subtilisin-chymotrypsin inhibitor-2A; WIP1, wound-induced protease inhibitor 1.


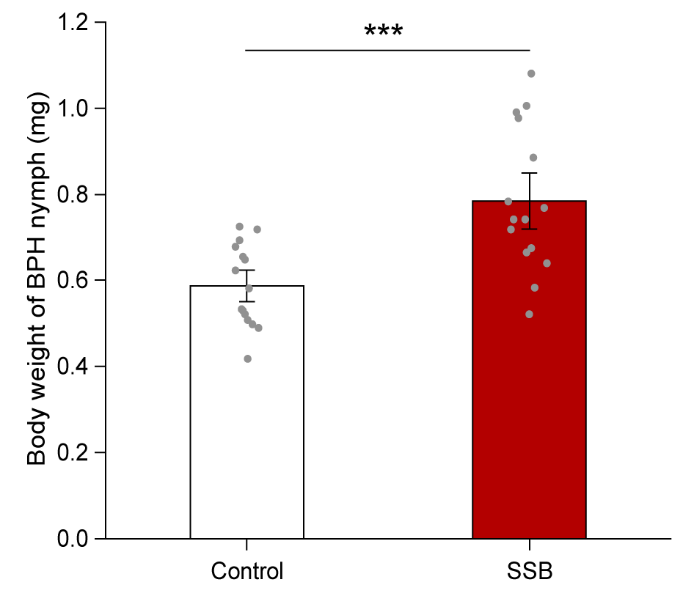


**Supplementary Figure 3** **Body weight of brown planthopper nymphs.** For rice striped stem borer (SSB) infestation treatment, rice plant was artificially infested with one 3^rd^ instar SSB larvae that had been starved for > 3 hr for 48 hr. Rice plants remained intact without insect infestation were set as control. SSB-infested or control plants were then infested with 10 gravid brown planthopper (BPH) female adults and allowed for free oviposition. After 24 hr, the BPH females were removed, and the plants were kept in climatic chambers until nymph hatched. Twenty new hatched BPH nymphs were transfered to another batch of SSB-infested or control plants. Eight days later, the number of BPH nymphs were checked and their body weight were weighed on a precision balance (CPA2250, Sartorius AG, Germany; readability = 0.01 mg). The mean weight of the nymphs on each plant was considered as one biological replicate, and 15 biological replicates were collected for each treatment. Exact *P* values = 4.66e-04. Data are presented as means ± SE. The body weight was analyzed using two-sided Student’s *t*-test (*** P< 0.001).


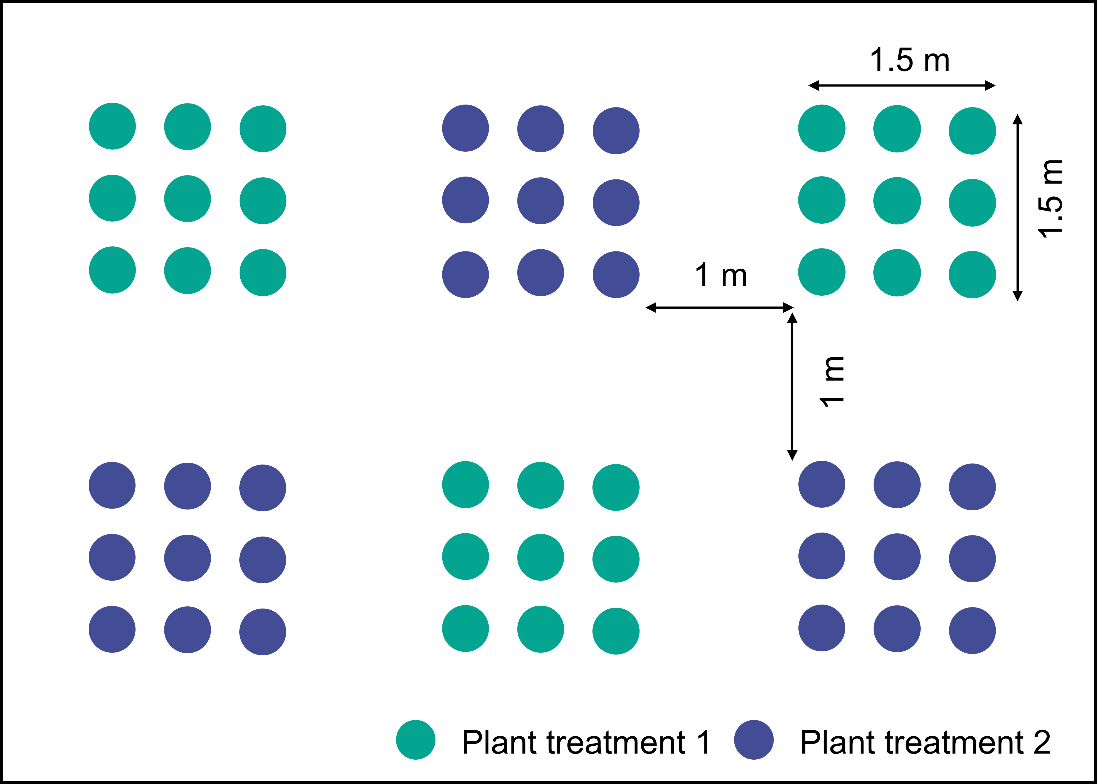


**Supplementary Figure 4** **Design for field cage experiment.** The treated rice plants were transplanted into experimental plots (1.5 m × 1.5 m). For each pairwise comparison, six plots of rice plants were covered with a screened cage (8 m × 5 m × 2.5 m) made of 80-mesh nylon net to prevent moths from entering or escaping. Each of the six plots contained 9 rice plants of a particular treatment, with 3 plots per cage representing the same treatment. Plots were separated by a 1-m buffer and they were alternately distributed in a 3 × 2 grid arrangement in each cage

**Supplementary Table 1 Genes and primer pairs used for quantitative real-time PCR analysis.**

| Gene ID | Gene name | Forward (F)/  Reverse (R) | Sequences (5’–3’) |
| --- | --- | --- | --- |
| Os01g0124200 | *APIP4* | F | CACCATGATCCGTCTCCCAAGC |
|  |  | R | CACTGCTTCACCTCGTCGTTGC |
| Os01g0124100 | *BBI3-2* | F | GGATTGCTGCGACAACACCA |
|  |  | R | CACCGGCTGGCAATCCTT |
| Os01g0124401 | *BBI3-3* | F | CTTCCACCATCCTGCTCTTCC |
|  |  | R | CACTGCTTCACCTCGTCGTT |
| Os01g0615050 | *OCPI1* | F | GCTAGTTTGCCGGACGATCA |
|  |  | R | TTGAGTGACGATGGTGCCTAC |
| Os01g0615100 | *OCPI2* | F | CATCAGACAGCAAGCCATGTG |
|  |  | R | CCAGAGGATGACACGGTTCG |
| Os03g0830600 | *PMEI15* | F | GTTCGAGAAGTTCATGCGGG |
|  |  | R | AGTTCGTCCGAGTGTTCATCAG |
| Os12g0437800 | *PR6* | F | CGCAGAGACGAGGGAAGATG |
|  |  | R | CGAATGGCAGCACGACGAT |
| Os12g0548401 | *SCI2A* | F | GAGGAGGCGAAGAAGGTGATC |
|  |  | R | GCGACGGTGTCAACGAAGA |
| Os01g0132000 | *WIP1* | F | AAATGCCGTGGGTGAGAGCAAG |
|  |  | R | GATGTCGTCGCAGGTGTAGAGC |
| Os01g0328400 | *Ubiquitin 5* | F | ACCACTTCGACCGCCACTACT |
|  |  | R | ACGCCTAAGCCTGCTGGTT |
