# Supplementary material for: Cooperative herbivory between two important pests of rice
Source: Nat Commun. 2021 Nov 19;12:6772. doi: 10.1038/s41467-021-27021-0 (PMC8604950; doi:10.1038/s41467-021-27021-0)
Supplement: Supplementary file 2 — Description of Additional Supplementary Files [file 41467_2021_27021_MOESM2_ESM.pdf]

Descriptions of Additional Supplementary Files

Supplementary Data 1
Description: Gene ontology analysis of the 992 downregulated genes between SSB/BPH and SSB infested plants.

Supplementary Data 2
Description: Orthologous Arabidopsis and rice genes used for Hormonometer analysis.

Supplementary Data 3
Description: Genes involved in JA biosynthesis that were activated by SSB feeding but were but were not induced by dual infestation.

Supplementary Data 4
Description: JA and SA-associated genes that were activated by SSB infestation, but were suppressed by dual infestation.

Supplementary Data 5
Description: TFs regulation prediction analyses of JA pathway genes.

Supplementary Data 6
Description: Volatiles released by differently infested rice plants. Volatiles cottected from the headspece of uninfested rice plants (Control), plants infested with 15 BPH nymphs mixed with 3rd and 4th instars for 48 hr (BPH), plants infested with one 3rd instar SSB larvae for 48 hr, plants simultaneously infested with one SSB larvae and 15 BPH nymphs for 48 hr (SSB/BPH).
